# Atrial Fibrillation and Aortic Ectasia as Complications of Primary Aldosteronism: Focus on Pathophysiological Aspects

**DOI:** 10.3390/ijms23042111

**Published:** 2022-02-14

**Authors:** Martina Bollati, Chiara Lopez, Fabio Bioletto, Federico Ponzetto, Ezio Ghigo, Mauro Maccario, Mirko Parasiliti-Caprino

**Affiliations:** Endocrinology, Diabetes and Metabolism, City of Health and Science University Hospital, Department of Medical Sciences, University of Turin, 10126 Turin, Italy; bollati.martina@gmail.com (M.B.); chiara.lopez@fastwebnet.it (C.L.); fabio.bioletto@unito.it (F.B.); federico.ponzetto@unito.it (F.P.); ezio.ghigo@unito.it (E.G.); mauro.maccario@unito.it (M.M.)

**Keywords:** primary aldosteronism, aldosterone, atrial fibrillation, aortic ectasia, cardiovascular risk, hypokalemia, adrenal glands, secondary hypertension, endocrine hypertension

## Abstract

Primary aldosteronism (PA) is the most common cause of secondary hypertension. A growing body of evidence has suggested that, beyond its well-known effects on blood pressure and electrolyte balance, aldosterone excess can exert pro-inflammatory, pro-oxidant and pro-fibrotic effects on the kidney, blood vessels and heart, leading to potentially harmful pathophysiological consequences. In clinical studies, PA has been associated with an increased risk of cardiovascular, cerebrovascular, renal and metabolic complication compared to essential hypertension, including atrial fibrillation (AF) and aortic ectasia. An increased prevalence of AF in patients with PA has been demonstrated in several clinical studies. Aldosterone excess seems to be involved in the pathogenesis of AF by inducing cardiac structural and electrical remodeling that in turn predisposes to arrhythmogenicity. The association between PA and aortic ectasia is less established, but several studies have demonstrated an effect of aldosterone on aortic stiffness, vascular smooth muscle cells and media composition that, in turn, might lead to an increased risk of aortic dilation and dissection. In this review, we focus on the current evidence regarding the potential role of aldosterone excess in the pathogenesis of AF and aortic ectasia.

## 1. Introduction

Primary aldosteronism (PA) is a clinical syndrome characterized by arterial hypertension (AH) and electrolyte imbalance due to an autonomous overproduction of aldosterone by one or both adrenal glands, independently of renin levels. Laparoscopic adrenalectomy is the treatment of choice in unilateral PA, whereas medical treatment with mineralocorticoid receptor (MR) antagonists (MRAs) represents the preferred option in bilateral forms [1,2].

PA is the most common cause of secondary hypertension. Its prevalence in hypertensive patients ranges between 5% and 15% in most studies [3,4] and increases with the severity of hypertension, reaching up to 29.1% in patients with resistant hypertension [5,6].

Beyond its well-known effect on sodium reabsorption in the distal nephron, a growing body of evidence has demonstrated that aldosterone levels that are inappropriate for salt intake can also exert pro-inflammatory, pro-oxidant and pro-fibrotic effects on the kidney, blood vessels and heart, leading to potentially harmful pathophysiological consequences [7].

**Figure 1:**
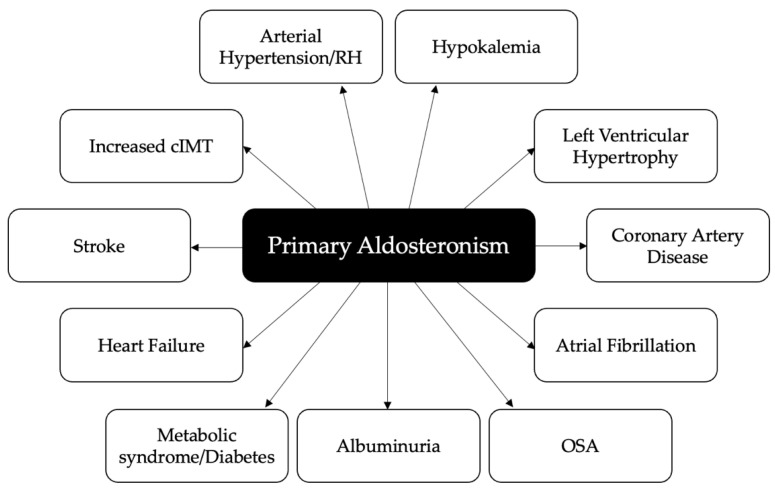
Cardiovascular, cerebrovascular, metabolic and renal complications associated with primary aldosteronism. RH: resistant hypertension; cIMT: carotid intima-media thickness; OSA: obstructive sleep apnea.

In clinical studies, PA has been associated with an increased risk of cerebrovascular events, myocardial infarction, left ventricular (LV) hypertrophy (LVH), atrial fibrillation (AF), increased carotid intima-media thickness, aortic ectasia, metabolic alterations and renal impairment as compared with essential hypertension (EH) [6,8,9] (Figure 1). An appropriate surgical or medical treatment of PA not only improves blood pressure control and corrects hypokalemia in most patients, but also seems to reverse target organ damage and reduce the increased risk of cardiovascular, cerebrovascular and renal complications [10,11,12,13,14,15].

In the present review, we focus on the potential role of aldosterone excess in the pathophysiology of AF and aortic ectasia.

## 2. Primary Aldosteronism and Atrial Fibrillation

AF is the most common sustained cardiac arrhythmia in adults and is associated with increased morbidity and mortality. Its prevalence increases with age and ranges between 2% and 4% in the adult general population [16].

AF is initiated by focal ectopic firing and is maintained by re-entry mechanisms in a vulnerable atrial substrate. The ectopic firing seems to arise from myocyte sleeves within the pulmonary veins and is triggered by a diastolic leak of Ca^2+^ from the sarcoplasmic reticulum that in turn determines myocyte depolarization due to an inward Na^+^ current via Na^+^-Ca^2+^ exchanger. The re-entry mechanism is promoted by slow conduction velocity of the depolarizing wavefront and a shortened refractory period of cardiac myocytes. The presence of structural and electrophysiological atrial abnormalities favors the self-perpetuation of AF by promoting re-entry [17].

In recent years, accumulating evidence has suggested a role for the renin-angiotensin-aldosterone system (RAAS) in the pathophysiology of AF and a potential association between AF and PA.

In a study by Goette et al., restoration of sinus rhythm by electrical cardioversion was associated with a fall in serum aldosterone in patients with persistent AF [18]. Similarly, in a prospective study conducted in 45 consecutive patients with non-valvular persistent AF and preserved LV systolic function, the authors found a positive correlation between the fall in aldosterone concentration 24 h after cardioversion and maintenance of sinus rhythm during a 30-day follow up [19].

In 2005, Milliez et al. reported a 12.1-fold greater risk of AF in 124 patients with PA as compared with 465 EH controls (7.3% vs. 0.6%); i multivariate analysis, PA remained an independent predictor of AF [20]. In a prospective study by Rossi et al. of 323 hypertensive patients who were systematically screened for PA, at baseline, patients with PA had a 7.2-fold greater prevalence of FA as compared to patients with EH (6.5% vs. 0.9%); at follow-up, the AF-free survival was significantly lower in the PA than in the EH group [11]. These data were confirmed in a meta-analysis by Monticone et al., who reported a 3.52-fold greater risk of AF in PA patients as compared to EH controls over a median follow up of 8.8 years [8].

On the other hand, in a population of 149 patients with a history of AF who were screened for PA using the aldosterone-to-renin ratio, a conclusive diagnosis of PA was made in 2.6% [21]. In a retrospective nationwide case-control study by the same group, the prevalence of PA in the AF group was relatively low (0.056%), but patients with AF had a significantly higher risk of being diagnosed with PA as compared with controls (OR 1.65). The low prevalence of PA reported in this study may be, at least in part, due to the retrospective design that might have led to an underestimation of PA cases in the absence of a systematic screening [22]. A much higher prevalence of PA was found in a prospective study recruiting 411 consecutive hypertensive patients with AF: PA was diagnosed in 42% of patients who showed no known cause of the arrhythmia, thus suggesting that PA is highly prevalent in hypertensive patients with unexplained AF [23].

Three different meta-analyses suggested a reduction in AF risk in MRA-treated patients as compared with controls, especially in the setting of heart failure (HF) [24,25,26]. As regards to patients with PA, in a large-scale prospective observational cohort study with an 11.8-year median follow-up, Rossi et al. reported a significantly worse AF-free survival in medically treated PA patients as compared with EH patients, whereas adrenalectomy was associated with AF-free survival similar to that of optimally treated EH patients, thus suggesting that removing the cause of aldosterone excess is better than controlling the MR-mediated effects of aldosterone [27].

Aldosterone has been shown to promote AF in animal models. Reil et al. demonstrated that the infusion of aldosterone in rats favors the development of AF after transesophageal atrial burst stimulation, independently of its effects on ventricular function or atrial pressure [28]. In another study by Lammers et al. in a rat model in which AF was induced by transesophageal burst pacing, aldosterone administration doubled the time until AF spontaneously converted into sinus rhythm [29].

On the other hand, Tsai et al. demonstrated that patients with AF had a significantly higher atrial MR expression as compared with those in sinus rhythm and that rapid depolarization increased MR expression through a Ca^2+^-dependent mechanism in HL-1 atrial myocytes, thus suggesting that AF itself can increase the sensitivity of cardiomyocytes to aldosterone [30]. Moreover, aldosterone itself has been shown to upregulate MR expression in cultured HL-1 cardiomyocytes, thus reinforcing its effects on the heart [31]. Since the MR not only binds aldosterone, but also cortisol, it can be argued that some effects mediated by MR activation may be attributable to cortisol. However, Lavall et al. reported that 11â-hydroxysteroid dehydrogenase type 2, an enzyme which converts cortisol to inactive metabolites allowing aldosterone binding to the MR, is upregulated in the left atrial myocardium of patients with AF, thereby suggesting that MR activation in this setting is mainly due to aldosterone [32].

**Figure 2:**
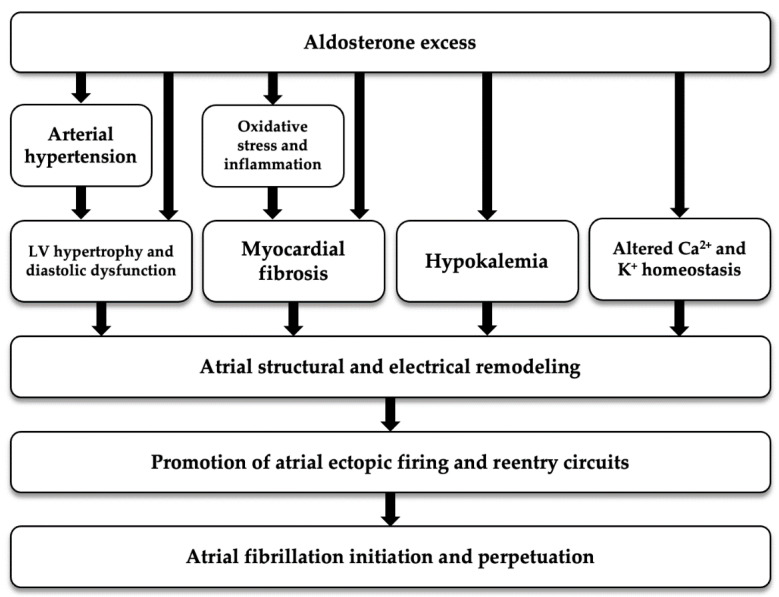
Potential pathogenetic mechanism linking aldosterone excess with atrial fibrillation. LV: left ventricular.

As regards to the potential physiopathological mechanism linking aldosterone excess to FA, aldosterone is thought to be involved in the genesis and perpetuation of AF not only by causing AH and electrolyte imbalance, but also by inducing inflammation, oxidative stress, fibrosis and electrophysiological changes; all these mechanisms contribute to structural and electrical atrial remodeling that are known to predispose to AF [33] (Figure 2).

### 2.1. Arterial Hypertension and Left Ventricular Hypertrophy

AH is a known risk factor for the development of AF [16]. AH causes LVH and diastolic dysfunction that in turn induce an increase in LV end-diastolic pressure and left atrial (LA) pressure, leading to atrial structural changes (dilation, hypertrophy, interstitial fibrosis, inflammatory infiltrates and apoptosis) with subsequent reduction and heterogeneity of conduction velocity [17]. Moreover, aldosterone induces LV remodeling and hypertrophy independently of its effects on blood pressure, since several studies have reported a greater increase in LV mass in patients with PA as compared with EH [8,11,34] probably because of direct pro-fibrotic, pro-oxidant and pro-inflammatory effects [35].

### 2.2. Hypokalemia

Hypokalemia is a common manifestation of PA, occurring in 9–37% of affected patients, and is a direct consequence of the activation of MR in the distal tubules and collecting duct of the nephron, where it mediates sodium reabsorption in exchange for potassium and hydrogen ions [2].

Hypokalemia itself can induce resting membrane hyperpolarization, Na^+^-K^+^ ATPase inhibition and suppression of K^+^ channel conductance, resulting in prolongation of the action potential duration, reduced repolarization reserve, early afterdepolarization, delayed afterdepolarization and automaticity, which may all participate in the genesis and perpetuation of AF [36].

### 2.3. Aldosterone and Atrial Fibrosis

As mentioned before, aldosterone is a well-known pro-fibrotic factor, and it exerts its effects both on the atria and on the ventricles [37].

Atrial fibrosis is a common finding in AF and is characterized by myocyte degeneration, fibroblast proliferation and interstitial expansion due to abnormal accumulation of fibrillar collagen, predisposing to arrhythmias by disrupting electrical conduction [38].

Sun et al. demonstrated that chronic administration of aldosterone to uninephrectomized rats in the presence of a high-salt diet leads to myocyte loss and interstitial collagen deposition in both right and left atria [39].

In a study by Lavall et al., aldosterone increased the expression of CTGF in vitro, probably by upregulating the RhoA/Rho kinase pathway via MR activation in cardiomyocytes. Overactivity of the RhoA/Rho kinase pathway has been observed in patients with aldosterone-producing adenomas and appears to be involved in the pathogenesis of arterial hypertension, by increasing peripheral vascular resistance via modulation of Ca^2+^ sensitivity in vascular smooth muscle cells (VSMCs) [40,41]. In the same study, aldosterone also increased the expression of miRNA-21, a microRNA that is associated with fibroblast survival and cardiac fibrosis, and lysyl oxidase, a key enzyme for collagen cross-linking, whereas the MR antagonists BR-4628 and spironolactone prevented these alterations [32].

In another study by Tsai et al. in HL-1 atrial cells, aldosterone mediated its fibrotic effects via MR and the MAPK intracellular signaling cascade, leading to an increase in the expression of collagen 1A and 3A, transforming growth factor (TGF)-â1 and á-smooth muscle actin (SMA) [31].

In a rat PA model, Reil et al. reported an increase in atrial fibroblasts and interstitial collagen, a reduction in active matrix metalloproteinase 13 and hypertrophy of atrial myocytes, as compared with controls [28].

MRAs have been shown to at least partially reverse aldosterone-induced atrial fibrosis in animal models. In a study by Milliez et al., the administration of spironolactone in the setting of HF induced a significant decrease in atrial fibrosis in a rat model [42]. These data were confirmed in a study by Yang et al. in congestive HF dogs, in which spironolactone reversed atrial fibrosis, decreased atrial refractory period and shortened conduction time [43]. Similarly, spironolactone treatment prevented arrhythmogenic atrial dilatation and reversed atrial fibrosis in a canine model of atrial fibrillation by normalizing the balance between matrix metalloproteinase (MMP)-9 and tissue inhibitors of metalloproteinase (TIMP)-1, whose alteration is associated with atrial fibrosis [44].

### 2.4. Aldosterone, Oxidative Stress and Inflammation

Increased oxidative stress and mitochondrial dysfunction have been reported in the atria of patients with AF. The main sources of oxidative stress in the atria are the mitochondrial electron transport chain, nicotinamide adenine dinucleotide phosphate (NADPH) oxidase, xanthine oxidase and uncoupled nitric oxide synthase (NOS) [45].

**Figure 3:**
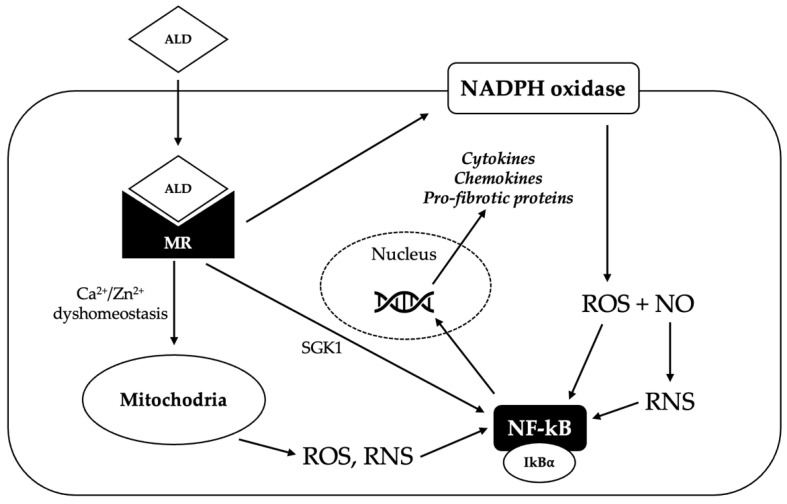
Potential mechanisms underlying the pro-oxidant effect of aldosterone. Aldosterone (ALD) diffuses through the plasma membrane to the cytosol, where it binds the mineralocorticoid receptor (MR), inducing its dimerization and activation. Activated MR promotes the generation of reactive oxygen species (ROS) via nicotinamide adenine dinucleotide phosphate (NADPH) oxidase; ROS react with nitric oxide (NO) to produce reactive nitrogen species (RNS). MR activation can also trigger the production of ROS and RNS in the mitochondria, probably by inducing Ca^2+^/Zn^2+^ dyshomeostasis. Increased ROS and RNS levels cause the release of nuclear factor kappa-light-chain-enhancer of activated B cells (NF-êB) from its repressor NF-êB inhibitor alpha (IkBá); NF-êB can therefore translocate in the nucleus, where it binds to a specific promoter and induces the transcription of pro-inflammatory and pro-fibrotic genes. Activated MR can also directly activate NF-êB, probably via the serum/glucocorticoid regulated kinase 1 (SGK1).

The pro-oxidant effect of aldosterone has been demonstrated in several studies, and oxidative stress seems to be an important cofactor in aldosterone-induced cardiac inflammation and fibrosis (Figure 3). In a study by Johar et al. in a rat model, the chronic infusion of aldosterone increased NADPH oxidase activity, as well as the expression of pro-fibrotic genes and nuclear factor kappa-light-chain-enhancer of activated B cells (NF-êB), inducing interstitial cardiac fibrosis [46]. In another study on a rat model by Sun et al., chronic exposure to aldosterone and salt was accompanied by a time-dependent sustained activation of NADPH oxidase with 3-nitrotyrosine generation and NF-êB activation in endothelial and inflammatory cells in the right and left ventricle, creating a pro-inflammatory and pro-fibrogenic environment [47].

Similarly, the administration of eplerenone in mice with chronic pressure overload was associated with a reduction in myocardial oxidative stress, as assessed by a reduction in 3-nitrotyrosine, as well as a diminished expression of intercellular adhesion molecule-1 and a reduced infiltration by macrophages [48].

In another a study by Kagiyama et al. in angiotensin II receptor knock-out mice, the exposure to aldosterone and salt increased cardiac expression of CTGF and NADPH components via the Rho kinase pathway, as well as the levels of nitrotyrosine and 4-hydroxy-2-nonenal, which are markers of oxidative damage. The administration of eplerenone antagonized these effects [49].

Moreover, in a study by Kamalov et al., aldosterone induced oxidative stress, as demonstrated by increased levels of 3-nitrotyrosine, 4-hydroxy-2-nonenal, malondialdehyde, H_2_O_2_ and oxidized glutathione, by inducing a dyshomeostasis of intracellular Ca^2+^ and Zn^2+^ in cardiac myocytes and mitochondria of rats [50].

Finally, aldosterone has been shown to induce Ca^2+^/calmodulin-dependent protein kinase II (CaMKII) oxidation via NADPH oxidase activation [51]. Oxidized CaMKII was found to be increased in atria from AF patients compared with patients in sinus rhythm [52].

Chronic inflammation, which is strictly related to oxidative stress, is another well-known pro-fibrotic factor and can contribute to the development of an AF-prone atrial substrate. Several studies have suggested a MR-mediated effect of aldosterone on inflammatory cell adhesion and infiltration in different tissues, such as the kidney, the vasculature and the adipose tissue [45].

As we have already mentioned, MR activation promotes inflammation by stimulating the generation of ROS, which in turn activate pro-inflammatory transcription factors such as NF-êB. Aldosterone has also been shown to directly activate the NF-êB pathway signaling in the kidney, leading to increased expression of several chemokines and interleukins, probably via the serum and glucocorticoid-inducible kinase 1 [53]. In addition to stimulating the formation of ROS, aldosterone may promote vascular inflammation by stimulating endothelial expression of adhesion molecules that, in turn, promote the recruitment of leukocytes [51].

As regards to the pro-inflammatory effects of aldosterone in the heart, Rocha et al. reported that the myocardial and coronary injury observed in angiotensin II/salt-treated rats, which was mainly inflammatory in nature and associated with the expression of cyclooxygenase 2 and osteopontin, was at least in part mediated by aldosterone, since treatment with eplerenone attenuated the cardiac and vascular damage [54].

### 2.5. Aldosterone and Electrical Remodeling

Abnormal Ca^2+^ handling is thought to be a possible mechanism underlying AF-generating ectopic foci [17]. Aldosterone has been shown to directly influence Ca^2+^ and K^+^ currents in cardiomyocytes in animal models, possibly participating in the electrophysiological changes underlying the development and perpetuation of cardiac arrhythmias. How the effects of aldosterone on Ca^2+^ and K^+^ currents might predispose to AF is, however, yet to be established.

In a study by Perrier et al. in a mouse model, elevated plasma aldosterone concentrations were associated with an increase in L-type channel Ca^2+^ current (I_Ca_) in ventricular cardiomyocytes, and this effect was attributable to changes in Ca^2+^ channel expression rather than modulation of channel activity [55]. These data were consistent with a previous study by Bénitah et al., in which aldosterone induced an increase in whole-cell I_Ca_ in isolated adult rat ventricular myocytes via the MR, probably by upregulating the expression of the á1c gene, which encodes for the á-subunit of the L-type voltage-dependent Ca^2+^ channel. The administration of spironolactone was able to blunt the aldosterone-induced increase in I_Ca_ density [56]. Similarly, in another study on cultured neonatal rat ventricular cardiomyocytes, aldosterone amplified L- and T-type I_Ca_, resulting in a positive chronotropic effect. This was associated with an increase in the mRNA coding for á1c and â2 subunits of cardiac L-type channel and an increased expression of T-type channel á-1H isoform. The administration of spironolactone inhibited the aldosterone-induced increase in beating frequency [57]. A more recent study by Mesquita et al. showed that aldosterone increases the expression of L-type channel á1c subunit N-terminal transcripts at both mRNA and protein levels through P1-promoter activation [58]. In the aforementioned study by Tsai et al. in HL-1 atrial myocytes, aldosterone increased expression of the á-1G and á-1H subunits of the T-type Ca^2+^ channel and thus increased the T-type I_Ca_ and intracellular Ca^2+^ load through a genomic pathway. In the same study, aldosterone decreased the rapidly activating delayed rectifier K^+^ current (I_Kr_), thereby altering repolarization [30]. In a study by Laszlo et al. in a rabbit model, the administration of spironolactone induced a decrease in L-type channel I_Ca_, but did not influence transient outward K^+^ current I_to_ in atrial myocytes [59]. In another study, Ouvrard-Pascaud et al. demonstrated that aldosterone upregulates L-type I_Ca_ and downregulates I_to_ in ventricular cardiomyocytes in a transgenic mouse model with cardiac-specific overexpression of the human MR, thereby prolonging the action potential and increasing the risk of arrhythmias [60].

Conversely, in a study in a rat model by Lammers et al., aldosterone infusion induced a significant shortening of the action potential in atrial cardiomyocytes. This was associated with an increased expression of K^+^ channel subunits K_ir_2.1 and K_v_1.5 that carry the inward rectifier K^+^ current I_k1_ and the ultra-rapid activating delayed rectifier K^+^ current I_kur_, respectively, both involved in the repolarization process. These effects were prevented by the administration of spironolactone [29]. The shortening of the action potential is associated with a reduction in the effective refractory period that, in turn, favors re-entry mechanisms underlying FA perpetuation [17].

Moreover, aldosterone has been shown to promote the occurrence of delayed afterdepolarization, a fundamental mechanism underlying several arrhythmias, by causing an abnormal diastolic opening of ryanodine receptors (RyR) that in turn determines an altered Ca^2+^ release from the sarcoplasmic reticulum in isolated adult rat ventricular cardiomyocytes. These changes were associated with downregulation of FKBP12 and FKBP12.6 that act as regulatory proteins of the RyR complex [61]. As we have already mentioned, the diastolic leak of Ca^2+^ from the sarcoplasmic reticulum activates an inward Na^+^ current via Na^+^-Ca^2+^ exchanger, resulting in spontaneous myocyte depolarization [17].

The slowing of conduction velocity, along with the shortening of the effective refractory period, contributes to the development of the re-entry mechanisms that underlie the perpetuation of AF [17]. In a study by Qu et al. in a rat model of cardiac hypertrophy, the administration of spironolactone reversed pathological gap junction remodeling and the associated slowing of impulse propagation, thereby improving conduction velocity, without influencing hypertrophy [62].

To summarize, aldosterone excess can contribute to the genesis and perpetuation of AF by inducing atrial structural and electrical remodeling. These consequences are not only dependent on the well-known influence of an aldosterone overproduction on blood pressure levels and K^+^ balance, but also on direct pro-oxidant, pro-inflammatory, pro-fibrotic and electrophysiological effects of aldosterone on cardiomyocytes.

## 3. Primary Aldosteronism and Aortic Ectasia

Aldosterone has been shown to have detrimental effects on blood vessels in the presence of a high salt intake, by inducing inflammation, oxidative stress, fibrosis and endothelial dysfunction [7].

The expression of MRs by aortic endothelial and VSMCs was reported almost 30 years ago [63]. However, there is little evidence on the association between PA and proximal aortic ectasia, a condition that is associated with an increased risk of aortic rupture and dissection [64].

In a rat model, the infusion of aldosterone and salt induced abdominal and thoracic aortic aneurysm formation and rupture in an age-dependent manner, whereas the administration of MRA significantly attenuated this effect [65].

In clinical studies, patients with PA have been shown to have larger ascending aortas and a higher prevalence of aortic ectasia as compared with patients with EH. In a prospective study on 45 patients with PA compared to 47 patients with EH, Zavatta et al. reported that patients with PA had larger ascending aortic diameters than those with EH before starting any specific treatment, even after considering possible confounding factors such as age, sex, body surface area, duration of hypertension, evidence of subclinical hypercortisolism and number of hypertensive drugs. After a mean follow-up of 3 years, patients with PA did not show significant changes in ascending aortic diameter, irrespective of the type of treatment (medical treatment with MRA vs. unilateral adrenalectomy), probably because a longer period of observation is necessary to detect significant differences [66]. In another study by Parasiliti-Caprino et al. in 110 patients with resistant hypertension, PA was associated with an increased prevalence of aortic ectasia as compared with patients with EH (22% vs. 6%), and this association remained statistically significant even in multivariate analysis, using age as a covariate [6]. In addition, proximal aortic aneurysm and aortic dissection have been described as potential complications in patients with PA in several case reports [67,68,69,70,71,72].

Measures of aortic stiffness, such as pulse wave velocity (PWV), have been shown to be independently associated with future thoracic aortic aneurysm expansion [73]. Several studies have reported that patients with PA have increased arterial stiffness, as indicated by higher values of PWV and augmentation index (AIx) as compared with patients with EH with comparable blood pressure levels [74,75]. Moreover, in patients with non-ischemic dilated cardiomyopathy, treatment with eplerenone induced a reduction of aortic stiffness, an increase in aortic distensibility and systolic aortic strain, independently of its effects on blood pressure [76]. Finally, aldosterone excess has also been associated with impaired endothelium-dependent vascular reactivity measured by flow-mediated dilation (FMD), which has been used as a surrogate marker of vascular health [40,77,78] and appears to be reduced in patients with ascending aorta dilation in the setting of Marfan syndrome and bicuspid aortic valve [79,80,81].

### 3.1. Effects of Aldosterone on Vascular Smooth Muscle Cells

In histological examination, ascending aortic aneurysm are characterized by focal loss of VSMCs, although areas of VSMCs hyperplasia that have been interpreted as an adaptive response to increased wall stress have also been reported [64].

Aldosterone has been shown to induce aortic VSMC apoptosis in rat models. A study by Yan et al. on a rat PA model showed that aldosterone can induce vascular cell apoptosis in vivo independently of blood pressure levels. This effect seems to be mediated by MR activation and an increased Bax/Bcl-2 ratio that induces apoptosis via the mitochondrial pathway [82]. Another study by Wei et al. showed that excess aldosterone promotes NADPH oxidase activation and reactive oxygen species production, which, in turn, impairs Akt serine phosphorylation and consequently causes activation of apoptotic signaling pathways, leading to vascular cell apoptosis in a rat model. MR antagonism with low-dose spironolactone prevented aldosterone-induced apoptosis by restoring Akt serine phosphorylation, independently of its effect on blood pressure [83].

Conversely, an in vitro study by Schwerdt et al. showed that increased cell loss in rat aorta VSMCs after exposure to aldosterone and salt reflected higher necrosis rates rather than higher apoptotic rates [84].

In contrast with the aforementioned evidence, other studies have shown that aldosterone promotes aortic VSMCs proliferation in rats [85].

### 3.2. Effects of Aldosterone on Extracellular Matrix

The histological examination of thoracic aortic aneurysms shows a maladaptive remodeling of the extracellular matrix, with disruption and loss of elastic fibers, accumulation of proteoglycans, altered collagen synthesis and fibrosis [64,86].

Several studies have investigated the effects of aldosterone on the composition of aortic extracellular matrix. In a study by Benetos et al. in spontaneously hypertensive rats, the administration of spironolactone prevented aortic collagen accumulation and reduced the collagen-to-elastin ratio, independent of blood pressure changes [87]. In another study by the same research group on old normotensive rats, the administration of spironolactone significantly decreased the collagen content in the carotid artery but not in the thoracic aorta [88]. Finally, the same authors reported that infusion of aldosterone in salt-drinking rats increased aortic stiffness, with elevated fibronectin but no change in collagen density [89].

In another study on a rat model, Iglarz et al. reported that a 6-week aldosterone infusion induced collagen deposition in the aortic media [90]. Similarly, Yan et al. showed that aldosterone can induce aortic fibrosis in a rat model by increasing the collagen production, probably via the upregulation of TGF-â1 expression. This profibrotic effect was prevented by MR antagonism with eplerenone, independently of blood pressure variations [82].

Moreover, Park et al. demonstrated that aortic collagen and the media cross-sectional area were significantly increased in aldosterone-infused rats and that the administration of an endothelin-1 (ET-1) type A receptor antagonist prevented these effects, thereby suggesting a role for ET-1 in fibrosis of large vessels in conditions associated with mineralocorticoid excess [91].

In a study by Harvey et al. on the abdominal aorta of stroke-prone spontaneously hypertensive rats, treatment with canrenoic acid blunted the MR-mediated activation of pro-inflammatory and pro-fibrotic signaling molecules (p66Shc and p38 MAPK) and prevented the expression of Nox1 and collagen I mRNA [92].

In another study on spontaneously hypertensive rats, the administration of eplerenone significantly reduced connective tissue growth factor (CTGF) mRNA expression in the aorta. In the same study, the authors also investigated the direct effect of aldosterone on VSMCs, showing that aldosterone increases CTGF production in a dose-related manner and that this effect is prevented by spironolactone [93].

Finally, an in vitro study by Gekle et al. in human aortic VSMCs reported that aldosterone, in combination with oxidative stress induced by H_2_O_2_, led to increased collagen synthesis that was reversed by MRA, leading to the idea that aldosterone sensitizes the cells to oxidative stress-induced collagen secretion [94].

In conclusion, aldosterone seems to modify the composition of the aortic extracellular matrix and to damage aortic VSMCs. These effects might alter the structural and functional properties of the aorta, thereby leading to increased arterial stiffness and weakening of the arterial wall, conditions that can in turn predispose to aortic enlargement and dissection.

## 4. Conclusions

In the past few decades, a growing body of evidence has suggested a detrimental effect of aldosterone excess on several target organs, such as the kidney, the vasculature and the heart. This is not only dependent on aldosterone influence on blood pressure levels, but also on its established pro-fibrotic, pro-inflammatory and pro-oxidant effects.

In clinical studies, PA has been associated with an increased risk of several cardiovascular, metabolic and renal complications as compared with EH, further validating this hypothesis. In this review, we summarized the current evidence on the potential role of aldosterone excess in AF and aortic ectasia.

An increased risk of AF has been demonstrated in patients with PA. As regards to the pathophysiological basis of this association, experimental studies have suggested a direct effect of aldosterone excess on cardiac structural and electrical remodeling, potentially leading to an AF-prone substrate. This association has some therapeutic implications, since some studies have demonstrated a beneficial role of MRAs on the risk of developing AF in the setting of HF. Concerning patients with PA, while adrenalectomy appears to be effective in reducing AF risk, further studies are needed to evaluate the role of MRAs.

Although evidence of the association of PA with aortic ectasia is limited, in clinical studies, patients with PA have been shown to have larger ascending aortas and a higher prevalence of proximal aortic ectasia as compared with patients with EH. Preclinical studies have demonstrated a direct effect of aldosterone on aortic VSMCs, media composition and wall stiffness, that in turn might lead to aortic wall weakening, dilation and increased risk of dissection. However, further research is needed to validate this hypothesis and to clarify if MR antagonism or surgical treatment may have a protective role in proximal aortic enlargement in patients with PA.

In conclusion, in patients with PA, aldosterone-mediated organ damage seems to be more extensive than previously thought, requiring a more holistic approach in the diagnosis and management of this disease and its potential complications.

## Data Availability

Not applicable.
